# The Genomic Capabilities of Microbial Communities Track Seasonal Variation in Environmental Conditions of Arctic Lagoons

**DOI:** 10.3389/fmicb.2021.601901

**Published:** 2021-02-12

**Authors:** Kristina D. Baker, Colleen T. E. Kellogg, James W. McClelland, Kenneth H. Dunton, Byron C. Crump

**Affiliations:** ^1^Department of Microbiology, Oregon State University, Corvallis, OR, United States; ^2^Hakai Institute, Heriot Bay, BC, Canada; ^3^The University of Texas at Austin Marine Science Institute, Port Aransas, TX, United States; ^4^College of Earth, Ocean, and Atmospheric Sciences, Oregon State University, Corvallis, OR, United States

**Keywords:** estuary, archaea, bacteria, omics, arctic national wildlife refuge

## Abstract

In contrast to temperate systems, Arctic lagoons that span the Alaska Beaufort Sea coast face extreme seasonality. Nine months of ice cover up to ∼1.7 m thick is followed by a spring thaw that introduces an enormous pulse of freshwater, nutrients, and organic matter into these lagoons over a relatively brief 2–3 week period. Prokaryotic communities link these subsidies to lagoon food webs through nutrient uptake, heterotrophic production, and other biogeochemical processes, but little is known about how the genomic capabilities of these communities respond to seasonal variability. Replicate water samples from two lagoons and one coastal site near Kaktovik, AK were collected in April (full ice cover), June (ice break up), and August (open water) to represent winter, spring, and summer, respectively. Samples were size fractionated to distinguish free-living and particle-attached microbial communities. Multivariate analysis of metagenomes indicated that seasonal variability in gene abundances was greater than variability between size fractions and sites, and that June differed significantly from the other months. Spring (June) gene abundances reflected the high input of watershed-sourced nutrients and organic matter via spring thaw, featuring indicator genes for denitrification possibly linked to greater organic carbon availability, and genes for processing phytoplankton-derived organic matter associated with spring blooms. Summer featured fewer indicator genes, but had increased abundances of anoxygenic photosynthesis genes, possibly associated with elevated light availability. Winter (April) gene abundances suggested low energy inputs and autotrophic bacterial metabolism, featuring indicator genes for chemoautotrophic carbon fixation, methane metabolism, and nitrification. Winter indicator genes for nitrification belonged to Thaumarchaeota and Nitrosomonadales, suggesting these organisms play an important role in oxidizing ammonium during the under-ice period. This study shows that high latitude estuarine microbial assemblages shift metabolic capabilities as they change phylogenetic composition between these extreme seasons, providing evidence that these communities may be resilient to large hydrological events in a rapidly changing Arctic.

## Introduction

The northeastern coast of Alaska is lined with a series of shallow lagoons that are protected from the Beaufort Sea by barrier islands. Of the 1,957 km of coastline along the Alaskan Beaufort Sea coast, 546 km are classified as lagoons with barrier islands (Jorgenson and Brown, 2005). These lagoons are home to a wide array of wildlife such as anadromous and marine fish, and birds from six continents that nest in the summer. They contain a diverse benthic community including bivalves, isopods, gastropods, and mysids. The lagoon food webs possess a high degree of omnivory and rely on terrestrial carbon as an energy source (Dunton et al., 2006; Harris et al., 2018).

The lagoons experience extreme shifts in seasonal environmental conditions, such as nine months of ice cover, water column temperatures ranging from −2 to 14°C, and salinity ranging from 0 to > 45 PSU (Harris et al., 2017). The spring freshet occurs as snowmelt begins and rivers thaw, resulting in over half of the annual freshwater river discharge occurring within a two-week period, usually in late May to early June (McClelland et al., 2014). This freshening not only changes the salinity of the lagoons but introduces terrestrially derived organic matter in dissolved and particulate forms. Terrestrial particulate organic matter (POM) is an important carbon source for the lagoons throughout the year, however, the composition of POM changes seasonally from relatively labile terrestrial material in the spring to more processed, refractory POM in winter (Connelly et al., 2015). Generally, dissolved organic carbon (DOC) is the most abundant form of terrestrial carbon transported by Arctic rivers (Gordeev et al., 1996; Lobbes et al., 2000; Holmes et al., 2012). In spring, this DOC is leached by snowmelt from frozen surface soils and plant litter and is more labile than DOC exported later in the summer (Holmes et al., 2008), likely because low temperature and rapid transport of this material off the frozen land surface limits terrestrial microbial processing. In summer, river DOC is less labile because warmer waters and longer travel times within river networks support greater microbial processing and photodegradation before this material makes it to the ocean (Holmes et al., 2008; Cory et al., 2014).

Previous work studying the food webs in these lagoons shows that organic matter in sediments and the water column have strong terrestrial signatures (Dunton et al., 2012; Harris et al., 2018). Terrestrially derived organic carbon is an important energy subsidy to members of lagoon food webs, especially some deposit feeders and omnivorous fishes who derive > 40% of their diet from terrestrial carbon (Harris et al., 2018). This subsidy is very high because the most abundant prey organisms in these systems, omnivorous detritivores, strongly rely on microbially processed terrestrial carbon (Dunton et al., 2012). Previous work also suggests that sediment microbial biogeochemical processes play a significant role in metazoan consumers assimilating terrestrial carbon (McTigue and Dunton, 2014). An absence of taxa in trophic positions between carbon sources and low-level consumers in the eastern Beaufort Sea suggest that microbes occupy this gap by processing terrestrial organic matter used by higher trophic levels (Bell et al., 2016). Thus, microbes may be a key component linking lagoon food webs to their major source of organic carbon.

Several studies have characterized Arctic microbial communities in freshwater systems and offshore environments (e.g., Garneau et al., 2006; Alonso-Sáez et al., 2008; Vallières et al., 2008; Galand et al., 2009; Underwood et al., 2019), but few studies have focused on the microbial communities of the nearshore Beaufort Sea where inputs of terrestrial material are very high. Changes in microbial community composition have been linked to seasonal variations in physical conditions and water chemistry in the coastal Beaufort Sea based on 16S and 18S rRNA gene data (Kellogg et al., 2019). The microbial community in the under-ice conditions in April were composed of coastal marine and chemoautotrophic taxa that potentially use methane, nitrogen, iron, and sulfur for energy during times when inputs of new/fresh organic matter are low. June communities were composed of freshwater and estuarine taxa, while August communities were composed of oligotrophic marine bacterial taxa (Kellogg et al., 2019).

Changes in microbial communities over seasons may in part be due to seasonal changes in the quantity and composition of dissolved organic matter (DOM). Sipler et al. (2017) found that additions of runoff from thawed permafrost to coastal water of the Chukchi Sea shifted the microbial community from oligotrophic to copiotrophic taxa. Approximately 7% of DOC and 38% of DON in these incubations were determined to be bioavailable within a 4–6 days time frame, showing that organic nutrients are labile and important sources to microbial communities (Sipler et al., 2017). Similarly, the molecular composition of DOM from melting first-year sea ice in the Canadian Arctic Archipelago has been shown to affect microbial community composition and abundance (Underwood et al., 2019). Microbial communities of varying size fractions, either particle-attached or free-living, can respond to changing environmental conditions differently (DeLong et al., 1993; Crump et al., 1999; Ghiglione et al., 2007; Garneau et al., 2009). Particle-attached communities from the Mackenzie River plume had higher activity in spring/summer than in fall/winter, and differences in particle-attached and free-living community composition and production were greatest during times of high POM input from the river (Garneau et al., 2009).

It is important to understand these microbial communities in the context of a changing Arctic climate. Longer periods of open water lead to longer growing seasons for phytoplankton, increasing the annual net primary production for these systems (Arrigo and van Dijken, 2015). Increased runoff of terrestrial organic matter in a warming Arctic may impact growth rates of heterotrophic bacteria, whose growth may be more significantly impacted by DOM than temperature (Peterson, 2002; McClelland et al., 2006; Déry et al., 2009; Kirchman et al., 2009; Rawlins et al., 2010; Romanovsky et al., 2010; Smith and Riseborough, 2010; Barnhart et al., 2014). Therefore, a clear gap in knowledge exists in understanding how marine microbial communities link terrestrial organic matter to the rest of the food web and how these linkages might be altered by a changing environment.

This study characterizes the metabolic capabilities of microbial communities across three seasons in two lagoons and one open coastal site along the eastern Alaska Beaufort Sea coast. We used metagenomic DNA sequence data of bacterial and archaeal water column communities to identify genes of relevant biogeochemical pathways. Results suggest that spring (June) and winter (April) months had the most strongly contrasting patterns in gene abundance. Spring had an increased abundance of genes involved in denitrification and processing of labile carbon from phytoplankton blooms, while winter had an increased abundance of genes involved in nitrification and chemoautotrophic carbon fixation. Summer (August) had few genes of elevated abundance relative to the other two seasons but featured the greatest abundance of genes for anoxygenic photosynthesis. These results suggest the potential for seasonal shifts in microbial community functions associated with different sources of nutrients and organic matter.

## Materials and Methods

### Study Site

**FIGURE 1:**
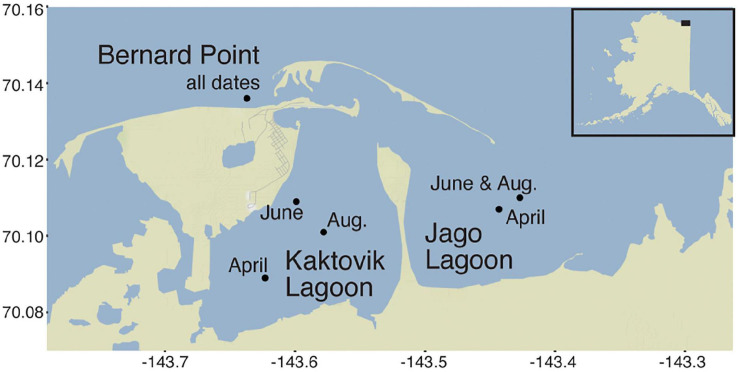
Map of sampling sites located near Kaktovik, Alaska. Three study sites are Bernard Point (BP), Jago Lagoon (JA), and Kaktovik Lagoon (KA).

Three sites located near the village of Kaktovik, Alaska were sampled during 2012. Kaktovik is an Iñupiat village located on Barter Island, along the northern-most part of the Arctic National Wildlife Refuge. The sample areas include two lagoons and one open coastal site (Figure 1). Jago Lagoon (JA; 70.106867, −143.443367) receives direct inputs from the Jago River and has direct connections to the Beaufort Sea through two wide inlets. Kaktovik Lagoon (KA; 70.089050, −143.623317) is a comparably smaller, more isolated system with no river input and has limited exchange with the Beaufort Sea owing to one narrow channel. The last site, Bernard Point (BP; 70.135617, −143.637367), is located along the northern shoreline of Barter Island in the Beaufort Sea.

### Sample Collection

Water samples were collected in April (full ice cover), June (ice break-up), and August (open water) 2012 at several locations within each lagoon and outside barrier islands (Figure 1). In April and June, a peristaltic pump was used to collect water 2 m below the surface or top of the ice. August samples were collected by submerging bottles by hand 0.5 m below the surface. Physical and biogeochemical parameters measured included particulate analysis (particulate organic carbon and nitrogen, chlorophyll *a*) as described in Connelly et al. (2015) and dissolved parameters (dissolved organic carbon and nitrogen, dissolved inorganic nitrogen) as described by the Beaufort Lagoon Ecosystems LTER and Core Program (2020a, b). Salinity, temperature, pH, and dissolved oxygen were measured using a sonde (YSI Corporation). Prokaryotic cell counts were measured by Kellogg et al. (2019). These environmental measurements are also discussed in Kellogg et al. (2019).

Within 6 h of collection, 4–11 L of water was first filtered through a 142 mm diameter, 3 micron pore size polycarbonate filter to collect particle-associated bacteria and then a 142 mm diameter, 0.22 micron pore size Supor filter (Pall) separated by a filter screen. Filters were placed into 15 mL falcon tubes and preserved with 8 mL of DNA extraction buffer (100 mM Tris, 100 mM NaEDTA, 100 mM phosphate buffer, 1.5 M NaCl, 1% CTAB) and frozen until extraction. Two replicates for each sample were collected from each site for a total of 36 samples (3 months, three sites, two size fractions, and replicates of each sample). One replicate BP June free-living sample was not processed due to contamination during transport.

### Environmental Sampling and Processing

Known quantities of *Thermus thermophilus* HB-8 DNA (purchased from ATCC) were added to samples prior to DNA extraction (10.0 ng *T. thermophilus* DNA per L of sample filtered) following Satinsky et al. (2013) as an internal control to account for DNA lost during extraction and sequencing. Filters then underwent phenol: chloroform extraction following Crump et al. (2003) but scaled up for larger starting volumes as described in Doherty et al. (2017). Library preparation and sequencing were carried out by the Center for Genomic Research and Biocomputing (CGRB) at Oregon State University, Corvallis, OR. Multiplexed, 150 basepair paired-end sequencing was performed on an Illumina HiSeq 2000.

### Metagenome Sequencing and Analysis

A detailed description of metagenome sequence analysis is provided in Supplementary Materials. Briefly, paired-end sequence reads were quality trimmed (BBDuk v.38.84, Bushnell, 2014), and internal *T. thermophilus* standard sequences were identified in a two-step process using BBSplit (BBTools; Bushnell, 2014) with a reference database of the *T. thermophilus* HB-8 genome, and with Kaiju (Menzel et al., 2016) with a reference database built using RefSeq (O’Leary et al., 2016). *T. thermophilus* reads were removed from the dataset, and the remaining reads for each sample were then assembled individually with metaSPAdes using default settings (v 3.11.0; Nurk et al., 2017). *T. thermophilus* sequences in each sample were enumerated (forward + reverse) and used to determine multipliers to calculate genes per liter according to Satinsky et al. (2013) (Supplementary Dataset 11, metadata tab). Briefly, the multipliers for each sample were calculated as Sa/(Sr * volume filtered) in which Sa is the number of molecules of *T. thermophilus* genomes added to the sample, and Sr is the number of *T. thermophilus* genomes recovered. Sr was calculated by dividing the number of *T. thermophilus* sequences recovered by the number of genes in the *T. thermophilus* genome (2,173).

Contig files for all samples were concatenated and dereplicated, and contigs less than 200 nucleotides in length were removed (Dedupe; Gregg and Eder, 2019). Concatenated contig sequences were submitted to IMG-MER^1^ for annotation with the DOE-JGI Metagenome Annotation Pipeline (Huntemann et al., 2016). After annotation, coding sequences (i.e., CDS or sequences coding for genes) were extracted from contigs to produce a Bowtie2 database of CDS sequences (Langmead and Salzberg, 2012). The quality-controlled sequences that were used for assembly (not including *T. thermophilus* sequences) were mapped to this database, and read counts were divided into separate files based on domain (Bacteria, Archaea, Eukaryote, Virus). For this study, only the Bacteria and Archaea sequences were further analyzed (see Supplementary Figure S1 for general workflow).

Read counts for each CDS were normalized following Wagner et al. (2012) for relative abundance and were also used to calculate absolute gene abundance following Satinsky et al. (2013). Briefly, the read count for each CDS was corrected for gene length and read length. To calculate genes per million (GPM) relative abundance for each CDS, this corrected read count (Tg) was scaled to 1 million [GPM = Tg * (1 × 10^9^/ΣTg)]. To calculate genes per liter absolute abundance, Tg was scaled to the original sum of mapped reads [Tg_scaled = Tg * (ΣRg/ΣTg)], and then multiplied by the *T. thermophilus* multiplier for that sample (see above). Then for each of these measures of abundance we summed abundances for CDS assigned to the same KO number and to the same phylodist string (Supplementary Table S1). Metagenome sequences were deposited in NCBI Sequence Read Archive (SRA) bioproject accession number PRJNA642637 under accessions SRR12147740-SRR12147774.

### Statistical Analyses

Environmental data were standardized using the range method and then log transformed using the biostats.R package in R (McGarigal, 2015). The transformed data were then analyzed with Principal Component Analysis (PCA) using the ggfortify package in R (Horikoshi and Tang, 2016; Tang et al., 2016), and plotted with the environmental variables shown as vectors.

To visualize microbial beta-diversity, a Bray-Curtis dissimilarity matrix was calculated from the table of gene abundances (GPM) and used in a non-metric multidimensional scaling (NMDS) analysis using the vegan package in R (Oksanen et al., 2019). Permutational multivariate analysis (PERMANOVA) was performed using the adonis function (999 permutations) from the vegan package in R to determine the variance in gene abundance associated with site, month, and fraction.

Percent relative abundances for taxa were plotted using ggplot2 (v2.2.1) with a cutoff at the class taxonomic level (Wickham, 2016). Abundance counts for replicate samples were averaged together. Counts per sample were normalized to percent relative abundance. Taxa with relative abundances less than 0.01 were classified as “other.”

The DESeq2 package in R was used to find significantly differentially abundant genes between the seasons (Love et al., 2014). While DESeq2 is commonly used in metatranscriptomics studies to analyze RNA sequence data, it has been shown to be a suitable method for identifying differentially abundant genes in metagenomic data (Dugat-Bony et al., 2015; Johnston et al., 2016; Jonsson et al., 2016). Gene counts were modeled following a negative binomial distribution. A likelihood ratio test was used as a hypothesis test, using a full and reduced model. The full model included the terms month and fraction, while the reduced model only included month to test if the fraction term increases the likelihood of the data. The likelihood ratio test is preferable when multiple interaction terms are being tested at once. False discovery rate (FDR) was controlled for using the Benjamini-Hochberg procedure with an alpha cutoff of 0.05. We further filtered CDS by using an adjusted *p*-value cutoff of < 0.05. Significant samples were transformed using variance stabilized transformation for visualization.

The log2fold changes of marker genes for carbon and nitrogen metabolic processes were assessed in order to look for variability in the potential for several nutrient cycling metabolic processes among months and size fractions. Previously identified marker genes, whose abundance can be used to assess the genetic potential of key steps in biogeochemical pathways, were identified in the DESeq2 output (Lauro et al., 2011; Llorens-Marès et al., 2015; Vavourakis et al., 2016; Kieft et al., 2018). Log2fold changes were plotted using the pheatmap package in R for the six different paired month comparisons. Overall, 47 different KO genes were used to assess 13 different metabolic pathways.

The function multipatt (multi-level pattern analysis) in the indicspecies package available through R (De Cáceres and Legendre, 2009) was used on the GPM gene abundances of only genes identified as significant by DESeq2 to identify significant indicator genes for each month. The setting delug = TRUE was used to limit analyses to single month combinations. This function compares association values between a gene and each of the 3 months, assigning a gene to the month with the highest association value. A permutation test was then performed to test the statistical significance of each relationship. The significant genes (*p* < 0.05) were analyzed with KEGG mapper to enumerate indicator genes present in KEGG pathways (Kanehisa and Sato, 2019). Only pathways featuring 10 or more indicator genes were analyzed, excluding several global and overview pathways. Pathways of interest were further analyzed by visualizing both relative abundance and genes/L abundance for each indicator gene in the pathway.

## Results

### Environmental Parameters

**FIGURE 2:**
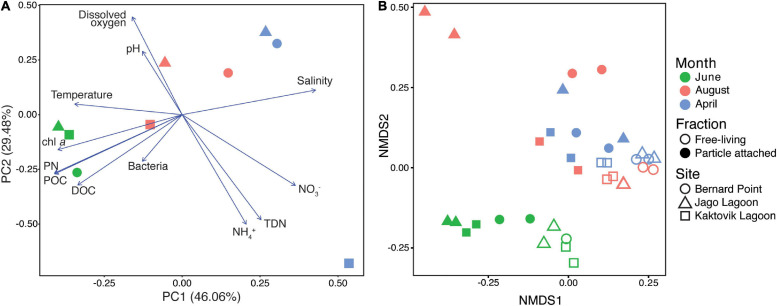
**(A)** Principal component analysis plot of environmental measurements, and **(B)** non-metric multidimensional scaling plot of gene per million (GPM) data for KEGG-annotated protein coding genes. Stress of NMDS is 0.085.

Principal component analysis of environmental data shows that samples cluster together by month on a horizontal axis where the primary axis explained 46% of the variation (Figure 2A), reflecting the physicochemical characteristics of the samples. Salinity and nitrate concentrations were highest in April, while chlorophyll, particulate and dissolved organic carbon, particulate nitrogen, and prokaryotic cell counts were highest in June. Water column temperature, however, is not clearly described in this analysis. Water temperature at the lagoon sites (KA and JA) was warmest in August (KA: 11.53°C, JA: 8.27°C), and water temperature at the BP site was warmest in June (1.55°C) (Supplementary Table S2). The secondary axis explained 29% of the variation, appearing to be largely influenced by the April KA sample which had higher total dissolved nitrogen and NH_4_, and lower dissolved oxygen.

### Metagenome Dimension Reduction Analysis

For abundances of different genes detected in metagenome samples, month was the most significant factor explaining variation (PERMANOVA, *R*^2^ = 0.41, *p* < 0.001). Size fraction also explained a significant fraction of variation (*R*^2^ = 0.20, *p* < 0.001), but site was not significant (*R*^2^ = 0.07, *P* = 0.336). NMDS analysis of relative gene abundances showed a clear separation between June samples and the other 2 months (Figure 2B). The samples clustered together to a lesser extent based on size fraction. The NMDS had a stress of 0.085, and the range of the Bray-Curtis dissimilarity was 0.027–0.456.

### Taxonomic Composition

Together, genes annotated to Alphaproteobacteria, Flavobacteria, and Gammaproteobacteria composed over 50% of the reads that mapped to CDS in all samples except for June free-living samples in which genes annotated to Betaproteobacteria also composed a large percentage of CDS (Supplementary Figure S2). In April, the KA samples differed from other samples, containing fewer Alphaproteobacteria genes and more Flavobacteria and Gammaproteobacteria genes. April samples also contained the highest percentages of genes from Euryarchaeota (ranging from 2.0 to 8.7%) and Thaumarchaeota (ranging from 0.9 to 4.4%). June samples differed from other months, with fewer Alphaproteobacteria genes, more Actinobacteria genes in the free-living fraction, more Cyanobacteria genes in the particle-attached fraction, and more Betaproteobacteria genes overall. The JA August particle-attached sample contained a higher percentage of Flavobacteria genes compared to the other August particle-attached samples. In both April and August, the free-living fraction had higher percentages of Alphaproteobacteria genes. Particle-attached samples in August had high percentages of Clostridia genes, up to 9.1% in the BP sample. Note that these gene abundances were not corrected for genome size, so taxa with larger genomes may be overrepresented relative to cell abundance.

### Differential Gene Abundance and Indicator Analyses

**FIGURE 3:**
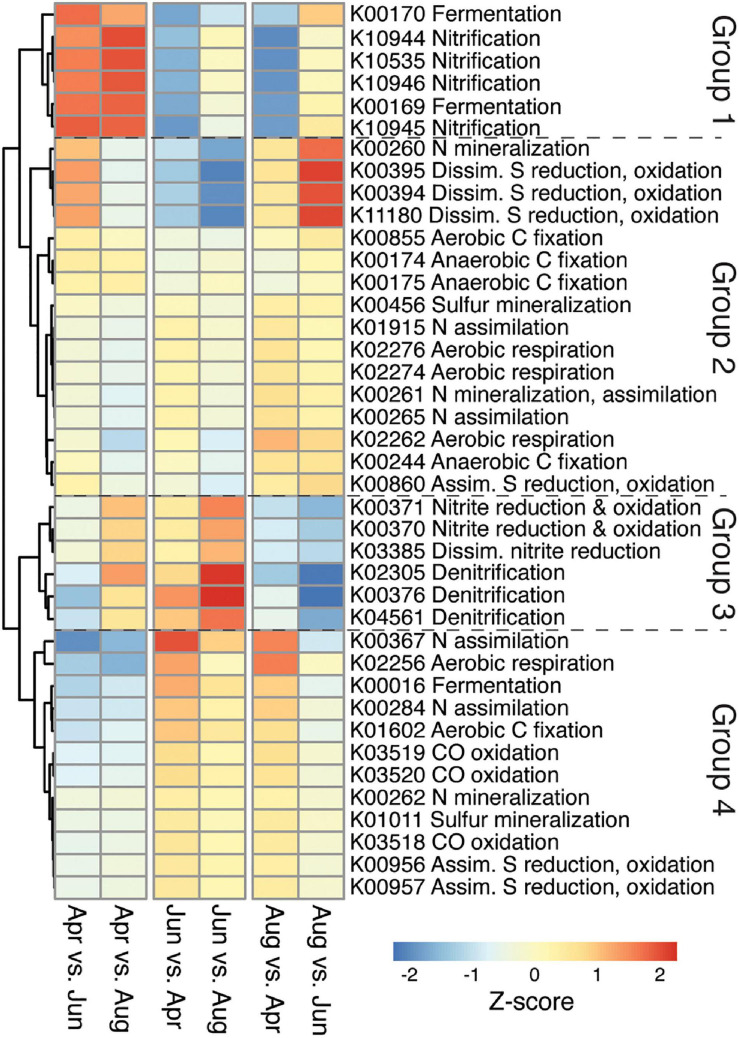
Heatmap of z-scaled log2fold change determined by DeSeq2 of marker genes for ecological functions. Log2fold change determined as month to month comparisons, i.e., first column “Apr vs. Jun” is April gene abundance as compared to June gene abundances. Red indicates higher abundance and blue indicates lower abundance.

DESeq2 identified 4,921 significant differentially abundant genes within the samples. Select marker genes, chosen based on previous studies (Lauro et al., 2011; Llorens-Marès et al., 2015; Hamilton et al., 2016; Kieft et al., 2018; Li et al., 2019; Shen et al., 2019), were used to identify seasonal shifts in genes involved in the nitrogen, carbon, and sulfur cycle (Figure 3). These genes formed four main groups. The first group included marker genes for fermentation and nitrification, that were more abundant in April than in June and August. The second group included marker genes for aerobic and anaerobic carbon fixation, aerobic respiration, organic nitrogen mineralization, inorganic nitrogen assimilation, and dissimilatory sulfate reduction/sulfide oxidation that were more abundant in April and August than in June. The third group included marker genes for denitrification, dissimilatory nitrate reduction, and nitrite oxidation and reduction that were more abundant in April and June than in August. The fourth group included marker genes for aerobic carbon fixation, aerobic respiration, carbon monoxide oxidation, fermentation, inorganic nitrogen assimilation, organic nitrogen mineralization, assimilatory sulfate reduction/oxidation, and sulfur mineralization that were more abundant in June and August than in April. Note that some marker genes used to detect the potential for anaerobic carbon fixation via the reductive TCA cycle (K00244, K00174, K00175) are not unique to that pathway and may participate in other metabolic processes.

**FIGURE 4:**
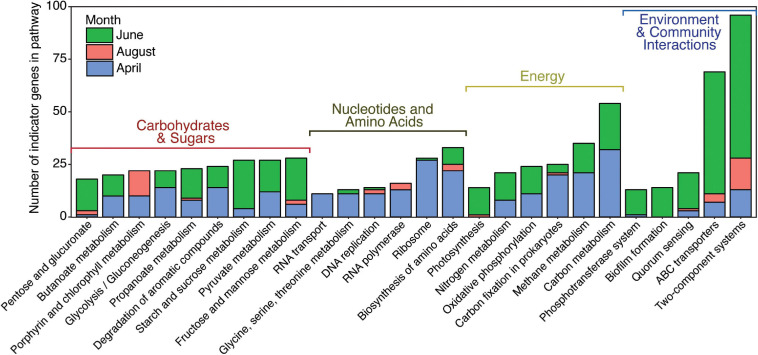
Number of indicator genes for each month grouped by KEGG pathways. Pathways that included more than 10 indicator genes for all 3 months are shown.

Indicator analysis found 4,113 genes that were indicators of month using a *p*-value of 0.05 and a test statistic cutoff of 0.8. Of these, 1,495 genes were indicators for April, 1,889 genes were indicators of June, and 729 genes were indicators of August. When classified into KEGG pathways, differences in gene diversity between months could be seen (Figure 4). Pathways were selected for further study based on their ability to provide insight into ecological function and archaeal abundance, using a cut-off of pathways with 10 or more indicator genes associated with a month. Broadly, these pathways categorize into four classes: energy metabolism, carbohydrate and other metabolism, nucleotide and amino acid biosynthesis and metabolism, and environmental and community interactions (Figure 4). This overview shows that the majority of genes that can be categorized to KEGG pathways are indicators for June and belong to processes such as energy and carbohydrate metabolism, as well as environmental interactions. A closer look at individual pathways revealed fine-scale shifts in indicator gene abundances between the months. Changes in individual gene abundances for nucleotide processing, the nitrogen cycle, two-component systems, and the ABC transporter system are described below.

Genes involved in nucleotide processing (DNA replication, RNA transport, and RNA polymerase) all had 11 or more indicator genes for April, with few indicator genes for the other months (Supplementary Table S3). Many of these indicator genes are specific to Archaea, such as RNA polymerase and parts of the DNA replication complex (Supplementary Figure S3). These archaeal nucleotide processing genes had abundances up to 1.6 × 10^9^ genes/L in the April free-living samples.

**FIGURE 5:**
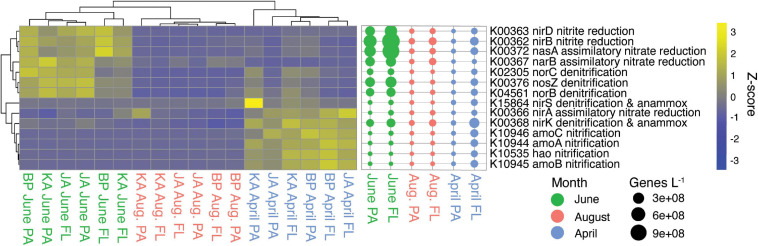
Nitrogen cycle indicator genes (KEGG pathway map00910). Heatmap of z-scaled GPM gene abundances for each sample with yellow being higher abundance and blue lower. Bubble plot of genes/L abundance for each month and size-fraction pairing.

The nitrogen cycle had a similar number of indicator genes for April and June, with June having 13 indicator genes and April having eight (Supplementary Table S3). April free-living samples had higher abundances of genes belonging to the nitrification process, while June samples (both fractions) had higher abundances of genes for denitrification and assimilatory and dissimilatory nitrate reduction (Figure 5).

**FIGURE 6:**
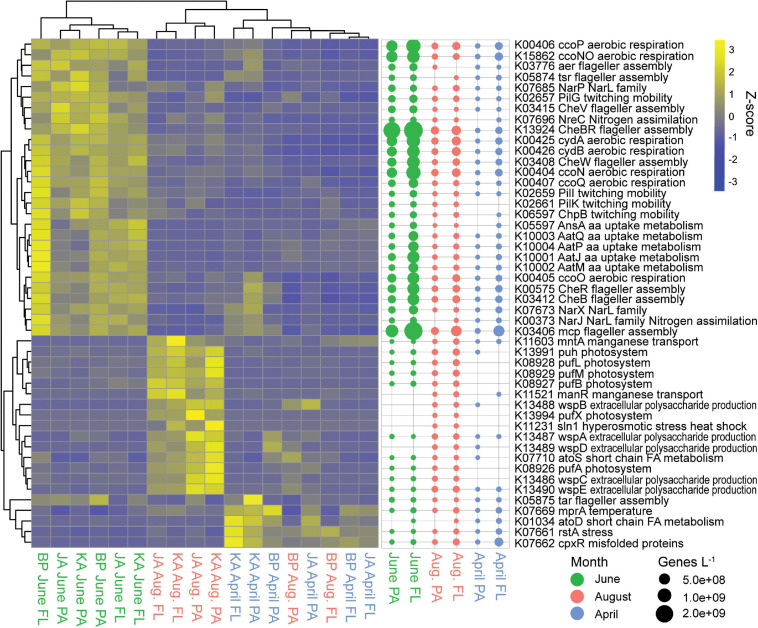
Two-component system indicator genes (KEGG pathway ko02020). Heatmap of z-scaled GPM gene abundances for each sample with yellow being higher abundance and blue lower. Bubble plot of genes/L abundance for each month and size-fraction pairing.

June had 68 indicator genes for two-component systems, while August had 15 and April had 13 (Supplementary Table S3). The June two-component system indicator genes were for cellular mobility, amino acid uptake and metabolism, nitrate/nitrite uptake, and aerobic respiration (Figure 6). Based on genes/L abundance, these genes were most abundant in the free-living June samples. Genes involved in aerobic respiration and flagellar assembly had the highest abundances, up to 1.2 × 10^9^ to 2.5 × 10^9^ genes/L, respectively. Genes involved in exopolysaccharide (EPS) production, manganese transport, and hyperosmotic stress/heat shock showed higher abundance in August estuarine (Jago and Kaktovik) samples. There were also six *pufX* photosystem genes that showed increased abundance in the August estuarine samples. All of these genes showed relatively low abundances in August (< 8.1 × 10^7^ genes/L) but were absent from many of the April and June samples. April and August coastal (Bernard Point) samples showed an increased abundance of genes involved in stress, temperature, and misfolded proteins, with free-living April samples having the highest abundances, up to 2.2 × 10^8^ genes/L.

June also had 58 indicator genes for the ABC transporter system, while April had seven and August had four (Supplementary Table S3). June particle-attached samples had higher abundances of capsular polysaccharide, lipopolysaccharide, xylitol, and s-methylcysteine transporter genes (Supplementary Figure S4). June free-living samples had higher abundances of cell-wall related transporter genes (e.g., teichoic acid and lipo-oligosaccharides), sugar transporters (e.g., erythritol, raffinose/stachyose/melibiose, and a-glucoside), glutamate/aspartate and biotin transporters, nitrogen transporters (e.g., nitrate/nitrite/cyanate), and sulfur transporters (e.g., alkanesulfonate, sulfate/thiosulfate). April samples had high abundances of indicator genes for the sugar transporter for α-1,4-digalacturonate, while August samples had one indicator gene each for vitamin B (biotin), lysine/arginine/ornithine/histidine, and manganese transporters.

## Discussion

The Arctic lagoons spanning the northern coast of Alaska support diverse food webs that are subject to extreme changes in environmental conditions among seasons (Dunton and Schell, 1987; Churchwell et al., 2015). While higher trophic level interactions have been studied in this region of the coastal Arctic Ocean (von Biela et al., 2011; Harwood et al., 2015), less is known about the role bacteria and archaea play in C and N cycling and the coastal food web (Boeuf et al., 2013; Kirchman et al., 2014; McTigue et al., 2016; Kellogg et al., 2019). Using shotgun metagenomics, this study found that the metabolic potential of coastal bacterial and archaeal communities is strongly linked to season, with June being the most distinct microbial community (Figure 2). June communities included genes involved in nitrate reduction and in acquiring and processing organic matter from phytoplankton and from terrestrial environments. April communities saw an increase in the abundance of Archaea along with ammonium oxidation and chemoautotrophic carbon fixation. August had fewer indicator genes, suggesting that, metabolically, the summertime open-water microbial community is a transitional community between the spring community and the winter community. To identify ecologically relevant variability in the genomic capabilities of lagoon microbial communities, we investigated differential gene abundance in KEGG pathways involving (a) two-component systems, (b) nucleotide biosynthesis, (c) nitrogen cycling, and (d) carbon cycling.

### Two-Component Systems

Two-component systems are signal-transduction pathways used by microbes to sense and respond to environmental stimuli (Hoch, 2000). June samples featured two-component system indicator genes associated with flagellar assembly and twitching mobility. These genes were largely from Alpha-, Beta-, and Gammaproteobacteria (Supplementary Table S4). Motility provides a fitness advantage to bacteria in environments that support strong gradients in resources such as those associated with phytoplankton blooms and the spring pulse of riverborne terrestrial material to the lagoons (Stocker and Seymour, 2012; Watteaux et al., 2015; Brumley et al., 2019). This is consistent with results from Tran et al. (2018) who found elevated expression of genes for flagella and chemotactic abilities by bacteria associated with phytoplankton blooms. Moreover, the high abundance of quorum sensing genes also found in June samples (Figure 4) may be linked to this higher abundance of motility genes, because motility and chemotaxis are partially regulated by quorum sensing (Sperandio et al., 2002; Zhou et al., 2016).

August samples featured only a small number of indicator genes, but these included several indicator genes for the *pufX* photosystem. *Puf* genes are commonly found in Rhodobacteraceae and code for light harvesting complex subunits used in aerobic anoxygenic photosynthesis (AAP), a photoheterotrophic process that harvests light for energy while still being capable of heterotrophy using organic carbon (Biel and Marrs, 1983; Zhu and Hearst, 1986; Farchaus et al., 1992). August indicator genes belonging to the *puf* operon were 60% Rhodobacteraceae when classified at the family level (Supplementary Table S5). An earlier study of 16S rRNA gene analysis at this study site found Rhodobacteraceae to be an indicator taxon for August samples (Kellogg et al., 2019). Rhodobacteraceae abundance has been linked to high nutrient conditions and phytoplankton blooms (González et al., 2000; Brown et al., 2005). One study showed that photoheterotrophic microbial communities off the coast of Utqiagvik, AK showed no change in relative abundance of *pufM* genes from winter to summer, however this study only looked at one gene and only sampled outside of the lagoons (Cottrell and Kirchman, 2009). *Puf* genes have also been found to be abundant in summer samples off the coast of Antarctica (Grzymski et al., 2012). Light has been found to enhance the abundance of AAPs (Ferrera et al., 2017), suggesting the open waters of August in the Beaufort lagoons allows this group of organisms to be successful.

### Nucleotide Processing

Genes from pathways relating to DNA and RNA processing were indicators for the month of April (Supplementary Figure S3), and a large portion of these are Archaea specific genes as identified by KEGG (Supplementary Figure S2). Most (up to 97.9%) of these indicator genes were annotated to Crenarchaeota, Euryarchaeota, and Thaumarchaeota, with Thaumarchaeota having the highest average GPM of 3.6 (Supplementary Table S6). Across all CDS, the percentage of genes belonging to Archaea vs. Bacteria in April was 6.4%, while it was < 1% for the other 2 months. Paralleling this trend, Thaumarchaeota had a > 1% cumulative relative abundance in April but < 1% abundance in the other months (Supplementary Figure S2). In the Eastern Beaufort Sea, Archaea comprised 16% of total cell counts in the winter but became undetectable in the summer (Alonso-Sáez et al., 2008). It is therefore likely that the archaeal indicator genes for DNA and RNA processing in April are a result of increased abundance of Archaea. Coastal archaeal communities in the Beaufort Sea are taxonomically distinct from river and offshore-marine communities and are dominated by Euryarchaeota, Crenarchaeota, and Thaumarchaeota (Galand et al., 2006). These coastal archaeal communities vary in composition depending on if they are particle-attached or free-living (Galand et al., 2008). Similarly, this study finds Thaumarchaeota to be the most abundant archaeal taxa, with higher abundances in the free-living fraction (Supplementary Figure S2). This study is among the first to compare changes in archaeal communities over multiple seasons in the coastal Arctic Ocean (Christman et al., 2011; Müller et al., 2018).

### Nitrogen Cycling

#### Nitrification

Nitrification genes including methane/ammonia monooxygenase (*pmo/amoABC*) and hydroxylamine dehydrogenase (*hao*) were indicators for the month of April, but KEGG does not distinguish between genes for *pmoABC* and *amoABC*. Across all samples, these genes belonged to nitrifying Nitrosomonadales (35%) and Thaumarchaeota (23%), and to methane oxidizing Methylococcales (41%), and the genes annotated to each of these groups were more abundant in April than the other months (Supplementary Table S7). The April peak in nitrification genes is partially explained by the increased relative abundance of Thaumarchaeota in April compared to other months (Supplementary Figure S2). However, Betaproteobacteria accounted for a large proportion of nitrification genes but showed the lowest relative abundance in April (Supplementary Figure S2). This suggests that there was a shift in Betaproteobacteria diversity during the winter toward organisms capable of nitrification. Higher abundance of the ammonia oxidizing organisms in April could be due to the higher levels of available nitrogen during this time of year. Several previous studies link seasonal increases in Thaumarchaeota abundance to elevated total nitrogen in the environment (Hollibaugh et al., 2011, 2014; Hong and Cho, 2015). Thaumarchaeota, which perform the first step of nitrification, have been found to become significantly enriched in under-ice communities of freshwater lakes (Butler et al., 2019), potentially playing a role in winter microbial biogeochemical cycling. The genus *Nitrosopumilus*, which accounts for most Archaea indicator genes for nucleotide processing (see above), are chemoautotrophs that release nitrogen-containing organic compounds and other labile DOM that may be limiting to heterotrophs in the community (Bayer et al., 2019).

Nitrification rates measured in the coastal Arctic Ocean range from 3.6 to 16 μmol N L^–1^ day^–1^, and nitrification accounts for the majority of ammonium uptake in the Arctic Ocean water column (Souza et al., 2014). The increased relative abundance of nitrification genes in winter is likely due to several controlling factors. First, ammonium concentration is highest in the lagoons in April (Kellogg et al., 2019). Second, phytoplankton abundance is lowest in the winter, thus ammonia oxidizing bacteria and archaea have less competition for ammonium since phytoplankton can typically outcompete ammonia oxidizing bacteria (AOB) and archaea (AOA) (Ward, 2005; Martens-Habbena et al., 2009). Third, light levels are reduced below ice in winter (Dunton, 1990), and nitrification can be inhibited by light (Ward et al., 1984; Souza et al., 2014). Fourth, nitrification rates in the coastal Chukchi Sea appear to be insensitive to changes in temperature (Baer et al., 2014) suggesting that the colder winter waters do not inhibit this process. One study in the coastal Chukchi and Beaufort Seas found that AOB and AOA *amoA* genes were 30- to 115-fold more abundant in winter compared to summer, mirroring higher potential nitrification rates in the winter as well as higher concentrations of available ammonium (Christman et al., 2011). Several other studies have also found potential nitrification rates in the Arctic to be higher in the winter (Baer et al., 2014; Souza et al., 2014). Nitrification rates have not been published for Arctic lagoons, but Connelly et al. (2014) found that the microbial community in these lagoons shifts from ammonium uptake in the summer to urea uptake in the winter. Sources of urea for the lagoons include riverine input, zooplankton excretion, melting seasonal ice, and sediment-associated bacteria (Connelly et al., 2014). Urea degradation has been found to enhance nitrification, suggesting that this urea is a potential source for nitrification in April (Swensen and Bakken, 1998; Sliekers et al., 2004).

#### Denitrification and Nitrate Reduction

Genes for nitrogen reduction were indicators for June including genes for nitrate reduction (*nasA*, *narB*), nitrite reduction (*nirB, nirD*), and denitrification (*norC, norB, norZ*). The genes *nasA* and *narB* are involved in nitrogen assimilation, while the genes *nirB* and *nirD* have both assimilative and dissimilative roles (Wang et al., 2019) including participation in the dissimilatory nitrate reduction to ammonia metabolism (DNRA; Heo et al., 2020; Figure 5). The typical marker gene for DNRA (*nrfA*, K03385; Pandey et al., 2020), though not identified as an indicator for June, was most abundant in June (Figure 3). There was also an elevated abundance of nitrate/nitrite ABC transporter genes in June, which could supply nitrate and nitrite for these metabolic processes (Supplementary Figure S4). The genes *nirB* and *nirD* were mainly associated with Gammaproteobacteria (55%) and Betaproteobacteria (19%), and the gene *nrfA* was associated with *Flavobacteriaceae* (61%), Gammaproteobacteria (22%), and Planctomycetes (17%). Genes for denitrification (*norC, norB, nosZ, nirS, nirK*) were mainly associated with *Flavobacteriaceae* (37%), Gammaproteobacteria (17%), and Alphaproteobacteria (17%) (Supplementary Tables S8, S9). Denitrification and DNRA are carried out by heterotrophic bacteria who use nitrate as an electron acceptor, but they require different sets of genes and have different end products: denitrification produces N_2_ gas while DNRA produces ammonium. Rates of denitrification and DNRA have not been published for the water column of Arctic lagoons, but previous studies in Arctic coastal sediments showed that sediment denitrification rates do not vary with season (Devol et al., 1997; Chang and Devol, 2009; McTigue et al., 2016). Similarly, a study in a Canadian lake found no change in water column denitrification rates between ice-covered and ice-free seasons (Cavaliere and Baulch, 2018). In contrast, our study suggests that the potential for water column denitrification and DNRA in the lagoons shows clear seasonality and peaks in June during the spring thaw.

When nitrate concentrations are high, availability of organic matter has been found to stimulate denitrification (Arango et al., 2007), and denitrification seems to be influenced by primary productivity and particulate organic carbon (Chang and Devol, 2009). In the Beaufort lagoons, the seasonal pulse of primary production by phytoplankton can reach the sediment before being remineralized, thus acting as a source of organic carbon for denitrification in both the water column and sediment (Sakshaug, 2004; Campbell et al., 2009; McTigue et al., 2016). While some previous studies have found evidence that anaerobic nitrogen processes can occur in the microhabitats of particle aggregates (Ganesh et al., 2014; Stief et al., 2016), other studies have found little difference in the contribution to denitrification between particle-attached and free-living microbial fractions (Donato et al., 2000; Chen et al., 2016). Consistent with this observation, we see little difference in abundance of denitrification genes between size fractions in June (within the same order of magnitude), suggesting that microbes capable of denitrification do not prefer one fraction (Figure 5).

### Carbon Cycling

The organic carbon supporting lagoon food webs enters the lagoons from multiple sources during June, when river discharge brings in terrestrial organic matter, diatom blooms fix carbon, and melting ice releases organic matter (Connelly et al., 2015; Traving et al., 2017; Kellogg et al., 2019). This large input of relatively labile organic carbon supports the food web through the rest of the year when inputs are lower and more recalcitrant. The composition and quality of organic matter (OM) changes seasonally with June having high proportions of terrestrial material and diatom production, August being dinoflagellate and/or green algae influenced, and April having low concentrations of predominantly refractory OM (Connelly et al., 2015). Microbial community composition and function often reflect seasonal variability in organic matter composition (Massana et al., 2001; Crump et al., 2003; Teeling et al., 2012; Sipler et al., 2017; Traving et al., 2017). We found similar results for Arctic lagoons in which the abundances of carbon-metabolizing genes followed seasonal patterns similar to those hypothesized to occur in Arctic freshwater lakes (Crump et al., 2003). According to this hypothesis, spring thaw introduces relatively labile terrestrial organic matter as well as nutrients to support phytoplankton production, and this OM supports elevated bacterial productivity. In August, the flux of terrestrial OM decreases and is less labile (Holmes et al., 2008), phytoplankton production slows (e.g., Chl *a*, Supplementary Table S2), and the microbial food web is supported by OM from degraded terrestrial material and from phytoplankton using recycled nutrients (Connelly et al., 2015; Kellogg et al., 2019). By April, labile components of OM are degraded, phytoplankton production is very low, and the microbial community is supported by more recalcitrant organic matter or alternate energy sources such as chemoautotrophy. We investigated genes involved in carbon fixation and the cycling of phytoplankton organic matter, terrestrially derived organic matter, and methane.

#### Carbon Fixation

Seasonal indicator genes associated with prokaryotic carbon fixation pathways were most abundant in April (Figure 4) when the carbon pool is dominated by relatively recalcitrant organic matter, and photosynthesis is low. Therefore, microbes must find a way to metabolize this material or find alternative energy pathways, such as chemoautotrophic carbon fixation (Connelly et al., 2015). Most of the April indicator genes associated with carbon fixation belong to pathways used by chemoautotrophic organisms (Supplementary Figure S5) including the hydroxypropiontate-hydroxybutyrate cycle (HHC), found in Thaumarcheaota and other autotrophic Archaea (Berg et al., 2007), and the reductive TCA cycle (rTCA), found in a broad diversity of autotrophic bacteria (Berg, 2011). Most of these genes are not unique identifiers for chemoautotrophic carbon fixation. However, six genes coding for HHC (K18602–K18605, K18593, K18594) were solely annotated to the phylum Thaumarchaeota (Supplementary Table 13), and four genes coding for rTCA (K00169–K00172) were mainly annotated to the phyla Nitrospirae and Nitrospinae (52%), suggesting that these genes are associated with chemoautotrophic nitrifiers. The other genes associated with these pathways were annotated to a broader array of taxa, but primarily to Group II Marine Euryarchaea and Thaumarchaeota.

April peaks in genes associated with chemoautotrophic carbon fixation supports Connelly et al. (2014) who hypothesized microbial dark carbon fixation processes occurring off the coast of Utquiavik, AK based on ^13^C stable isotope probe data. Genes for HHC have also been found to be abundant in winter coastal communities from Antarctica (Grzymski et al., 2012). In contrast, many of the June indicators for carbon fixation belong to the Calvin Cycle (Supplementary Figure S5), which is used by photosynthetic organisms including cyanobacteria. Most of the indicator genes that coded for the Calvin Cycle are not unique markers of this process, but one Calvin Cycle marker gene, ribulose-bisphosphate carboxylase (K01602), peaked in abundance in the particle-attached fraction in June (Supplementary Figure S2). This gene was annotated to a broad diversity of bacterial including Rhizobiales (38%) and Burkholderiales (23%), but very few cyanobacteria (1%) suggesting that this gene is not associated with photoautotrophs in the lagoons.

#### Phytoplankton Organic Matter

June communities had a greater number of indicator genes for photosynthesis, pentose and glucuronate interconversions, propanoate metabolism, starch and sucrose metabolism, and fructose and mannose metabolism compared to the other 2 months (Figure 4). The photosynthesis indicator genes were photosystems I and II, and cytochrome b_6_f complex genes belonging to Chroococcales, Nostocales, Oscillatoriales, and Synechococcales cyanobacteria (Supplementary Table S10). Cyanobacteria had a higher abundance in June particle-attached samples (Supplementary Figure S2), although Kellogg et al. (2019) did not identify cyanobacteria as a June indicator species in unfiltered samples. Previous studies found cyanobacterial abundance to be higher in Arctic rivers and to decrease with increasing salinity along the coast, suggesting they are tied to spring thaw and are allochthonous to the lagoons (Garneau et al., 2006; Waleron et al., 2007; Vallières et al., 2008).

June indicator genes for propanoate metabolism may be linked to the availability of dimethylsulfoniopropionate (DMSP), an organosulfur compound produced by phytoplankton as an osmolyte and possible cryoprotectant (Kirst et al., 1991). Bacterial degradation of DMSP leads to acryloyl-CoA (Curson et al., 2008; Todd et al., 2009) or methionine and cysteine (Kiene and Linn, 2000), and these C3 compounds can be further processed in the propanoate pathway. One fate of DMSP-derived C3 compounds is to be transformed into acetyl-coA, which is then taken to the TCA cycle (Vila-Costa et al., 2010). DMSP is in higher concentrations in polar marine environments than temperate and tropical regions (Lana et al., 2011), and is in very high concentrations in association with sea-ice algae (Galindo et al., 2014). In the Canadian Arctic, DMSP concentrations were correlated with chlorophyll-a concentrations (Luce et al., 2011), and DMSP released from thawing sea ice was found to be rapidly consumed by microbes (Galindo et al., 2014). Increased levels of DMSP have been shown to correlate with the transcription of propanoate metabolism pathway genes in marine microbial communities, suggesting that this pathway is important for DMSP degradation (Vila-Costa et al., 2010). These previous studies are consistent with our finding of abundant genes for propanoate metabolism in June when diatoms were abundant (Connelly et al., 2015; Kellogg et al., 2019) and sea ice was thawing and potentially releasing DMSP into the water column.

Besides DMSP, phytoplankton blooms also release a variety of other low molecular weight (LMW) compounds such as organic acids and sugar alcohols toward the beginning of blooms, and later release high molecular weight (HMW) compounds such as polysaccharides and lipids (Buchan et al., 2014). These compounds impact microbial communities because LMW compounds can be used by most heterotrophic bacteria, but HMW compounds are only available to a restricted set of organisms (Logue et al., 2016). June indicator genes in the ABC transporters pathway included genes for transport of many of these HMW and LMW compounds (Supplementary Figure S4). This is consistent with previous studies suggesting that ABC transporter genes are expressed in response to dissolved phytoplankton material and other DOC (Poretsky et al., 2010; Williams et al., 2013). High abundances of these genes in June as well as genes for starch and sucrose metabolism, fructose and mannose metabolism, and glucose and glucuronate interconversions (Figure 4) may be linked to production of these compounds by spring phytoplankton blooms.

#### Terrestrial Organic Matter

Terrestrial DOM supplied by Arctic rivers is mainly composed of lignin-like compounds, followed by protein, lipid, and unsaturated hydrocarbon-like compounds (Sipler et al., 2017). The process of microbially mediated lignin degradation is still not well understood (Brown and Chang, 2014; Datta et al., 2017). While there have been genes identified in bacterial lignin degradation, few are annotated to the KEGG database (Datta et al., 2017). However, we found that the dye-decolorizing peroxidase (DyP, EC 1.11.1.19, KO:K15733) gene was significantly differentially abundant, and was most abundant in June and August and close to zero abundance in April. This gene is important in the bacterial oxidation of lignin, and also acts on a broad range of substrates including phenols, hydroquinones, dyes, amines, aromatic alcohols, and xenobiotics (van Bloois et al., 2010; de Gonzalo et al., 2016; Datta et al., 2017). This gene belonged to taxa that were not detected in April metagenomes including the Actinobacteria families *Microbacteriaceae* and *Streptomycetaceae* (Supplementary Table S11). In contrast with *DyP*, most degradation genes for aromatic compounds were indicators for either June or April (Figure 4), with the difference being that April had more genes involved in the anaerobic conversions of aromatic rings (four in April vs. 1 in June). These results suggest that spring and summer microbial communities possess elevated potential to degrade lignin and aromatic components of the terrestrial OM delivered to in spring. By April, however, labile compounds likely become scarce, thus making aromatic compounds (commonly considered refractory) more favorable for microbial degradation. Overall, the degradation of large organic molecules is a complex process, and our results suggest taxa capable of different OM degradation pathways vary in abundance throughout the year in response to the available forms of terrestrial organic matter.

#### Methane Metabolism

Seasonal indicator genes belonging to the methane metabolism KEGG pathway were most abundant in April. Three of these genes code for methane/ammonia monooxygenase subunits (K10944–K10946; see section “Nitrification”) and were annotated to methane oxidizing Methylococcales (52%), and ammonia oxidizing Nitrosomonadales (31%) and Thaumarchaeota (26%) (Supplementary Table S12). Methylococcales abundance was elevated in Arctic lagoons in April (Kellogg et al., 2019), and one study off the coast of Utquiavik, AK found increased abundance of Gammaproteobacteria methane oxidizing bacteria (MOB) in April seawater and a strong correlation between methylotrophs and the methane oxidation rate constant (Uhlig et al., 2018). Methane concentrations along the Beaufort Shelf increase during periods of ice cover, ranging from 0.14 to 0.43 mg CH_4_ m^–2^ day^–1^ in ice-free conditions to 0.28–1.01 mg CH_4_ m^–2^ day^–1^ during ice-cover (Lorenson et al., 2016). Decomposing sedimentary organic matter is the source of this methane, and microbial degradation of the sedimentary organic matter is the main pathway by which methane is released into the water column (Lorenson et al., 2016). This suggests that further study of the sediment microbial community is needed to better understand methane cycling in this system.

## Conclusion

This study suggests that the water column microbial community in Arctic coastal lagoons plays a key role in processing both terrestrial and autochthonous organic matter throughout the year, though how it processes this material varies with season. These findings can inform future studies on Arctic lagoon food webs and the roles of microbes. This will be especially important in the future, because changes affecting seasonality such as rising temperature, changes in river discharge, freshening, and increase in open water season are expected to occur due to climate change (McClelland et al., 2006; Serreze and Barry, 2011; Morison et al., 2012; Barnhart et al., 2014). These changes can impact phytoplankton production, although various factors could increase or decrease production, and in turn alter organic matter availability and heterotrophic processes, likely altering the microbial community composition and function (Wassmann and Reigstad, 2011). Future work looking at the sediment microbial community as well as pairing metatranscriptomics with biogeochemical rate measurements would further our understanding of how microbes process organic matter and their connection to higher trophic levels.

## Data Availability Statement

The datasets presented in this study can be found in the NCBI Short Read Archive BioProject# PRJNA642637 under accessions SRR12147740–SRR12147774, https://www.ncbi.nlm.nih.gov/.

## Author Contributions

KB performed DNA sequence data analysis and lead writing. CK collected and processed samples, and conducted DNA sequencing. BC contributed to writing and interpretation. BC, JM, and KD conceptualized project. JM and KD led field efforts and collected samples. All authors contributed to manuscript revision, read, and approved the submitted version.

## Conflict of Interest

The authors declare that the research was conducted in the absence of any commercial or financial relationships that could be construed as a potential conflict of interest.

## Footnotes

1 https://img.jgi.doe.gov
