# Supplementary material for: The Genomic Capabilities of Microbial Communities Track Seasonal Variation in Environmental Conditions of Arctic Lagoons
Source: Front Microbiol. 2021 Feb 12;12:601901. doi: 10.3389/fmicb.2021.601901 (PMC7906997; doi:10.3389/fmicb.2021.601901)
Supplement: Supplementary file 1 [file Presentation_1.pdf]

Title
The genomic capabilities of microbial communities track seasonal variation in environmental conditions of Arctic lagoons

Authors
Kristina D. Baker[1], Colleen T. E. Kellogg[2], James W. McClelland[3], Kenneth H. Dunton[3], Byron C. Crump[4*]

Affiliations
[1] Department of Microbiology, Oregon State University, Corvallis, OR, 97331, USA
[2] Hakai Institute, Heriot Bay, BC, Canada,
[3] The University of Texas at Austin Marine Science Institute, Port Aransas, TX 78373, USA
[4] College of Earth, Ocean, and Atmospheric Sciences, Oregon State University, Corvallis, OR, 97221, USA

*Corresponding Author
College of Earth, Ocean, and Atmospheric Sciences
Oregon State University
104 CEOAS Admin Bldg.
Corvallis, OR 97331-5503
Phone: 541-737-4369-
Byron.Crump@oregonstate.edu

This PDF file includes:

Supplementary Methods
Supplementary Figures S1 to S5
Caption for Supplementary Tables S1 to S12
PERL scripts
References

Other supplementary materials for this manuscript include the following Excel files:

Supplementary Table S1
Supplementary Table S2
Supplementary Table S3
Supplementary Table S4-12

Supplementary Methods

Metagenome sequencing and analysis

Forward and reverse paired-end reads were quality trimmed and filtered using BBDuk (v. v.38.84; Bushnell 2014), where both ends of the reads were quality trimmed at a Phred score of Q=10 and reads less than 50 base pairs long (after trimming) were removed. Internal *T. thermophilus* standard sequences were identified in a two-step process. First, potential *T. thermophilus* reads were identified using the program BBSplit (BBTools; Bushnell 2014) and the genome sequence of *T. thermophilus*. BBSplit is generally used to remove contaminant sequences from datasets, and tends to identify false positives. Then the pool of potential *T. thermophilus* sequences was further classified with the program Kaiju (Menzel et al., 2016) using the options for greedy mode, allowing five substitutions, and a reference database built using RefSeq (O'Leary et al., 2016) plus the sequences of the reference *T. thermophilus* genome. Taxonomy names, including the full taxonomy path, were added to the Kaiju output using Kaiju's addTaxonNames, omitting unclassified reads (Menzel et al., 2016). Sequences classified as *Thermaceae* at the family level for either the forward or reverse reads (or both) were considered *T. thermophilus* reads. The Kaiju script filterbyname then removed *T. thermophilus* sequences from the quality controlled fastq sample files. The remaining reads for each sample were then assembled using metaSPAdes (v 3.11.0) using default settings (Nurk et al., 2017). Assemblies were assessed by mapping non-*T. thermophilus* reads to assembled contigs using Bowtie 2 (Langmead and Salzberg, 2012). SAMtools was used to view the .sam files and count the number of raw reads that mapped to the contigs (Li et al., 2009). *T. thermophilus* sequences in each sample were enumerated (forward + reverse) and used to determine multipliers to calculate genes per liter according to Satinsky et al. (2013) (Supplementary dataset 11, metadata tab). Briefly, the multipliers for each sample were calculated as Sa/(Sr * volume filtered) in which Sa is the number of molecules of *T. thermophilus* genomes added to the sample, and Sr is the number of *T. thermophilus* genomes recovered. Sr was calculated by dividing the number of *T. thermophilus* sequences recovered by the number of genes in the *T. thermophilus* genome (2173).

Contig files for all samples were concatenated into a single file and contigs less than 200 nucleotides in length were removed and the contigs were dereplicated using Dedupe (Gregg and Eder, 2019). Concatenated contig sequences were submitted to IMG-MER (https://img.jgi.doe.gov) for annotation with the DOE-JGI Metagenome Annotation Pipeline (Huntemann et al., 2016). After annotation, .gff files containing Phylodist and Kegg Orthology (KO) gene numbers were reformatted to include only CDS, or sequences coding for a gene (coding sequence). This was used along with the assembled contig fasta file to produce a fasta file of CDS sequences with a PERL script (contig_regions_new.pl; see below). Bowtie 2 was then used to create a database from this fasta file of CDS sequences, and then map the quality controlled fastq files without *T. thermophilus* sequences to the CDS database. Read counts were divided into separate files based on domain (Bacteria, Archaea, Eukaryote, Virus). A small number of mapped reads classified as *Thermaceae* at the family level were then separated out of the Bacteria reads. For this study, only the Bacteria and Archaea sequences were further analyzed (see **Supplementary Figure S1** for general workflow).

Read counts for each CDS were normalized following Wagner et al. (2012). Briefly, the number of sequences that mapped to each CDS, the CDS length, and the average length of mapped sequences were extracted from the CDS .sam file using pileup.sh (BBTools; Bushnell 2014). The abundance of each CDS was corrected for gene length and read length by multiplying

the number of mapped reads (Rg) by the average length of mapped reads, and dividing by the CDS length, resulting in the corrected read count (Tg). To calculate genes per million (GPM) relative abundance for each CDS, this corrected read count (Tg) was scaled to one million (GPM = Tg*($1x10^9/\Sigma Tg$)). To calculate genes per liter absolute abundance, Tg was scaled to the original sum of mapped reads (Tg-scaled = Tg *($\Sigma Rg/\Sigma Tg$)), and then multiplied by the *T. thermophilus* multiplier for that sample (see above). Then for each of these measures of abundance we summed abundances for CDS assigned to the same KO number and to the same phylodist string (**Supplementary Table S1**). Metagenome sequences were deposited in NCBI Sequence Read Archive (SRA) bioproject accession number PRJNA642637 under accessions SRR12147740-SRR12147774.

Supplementary Figures

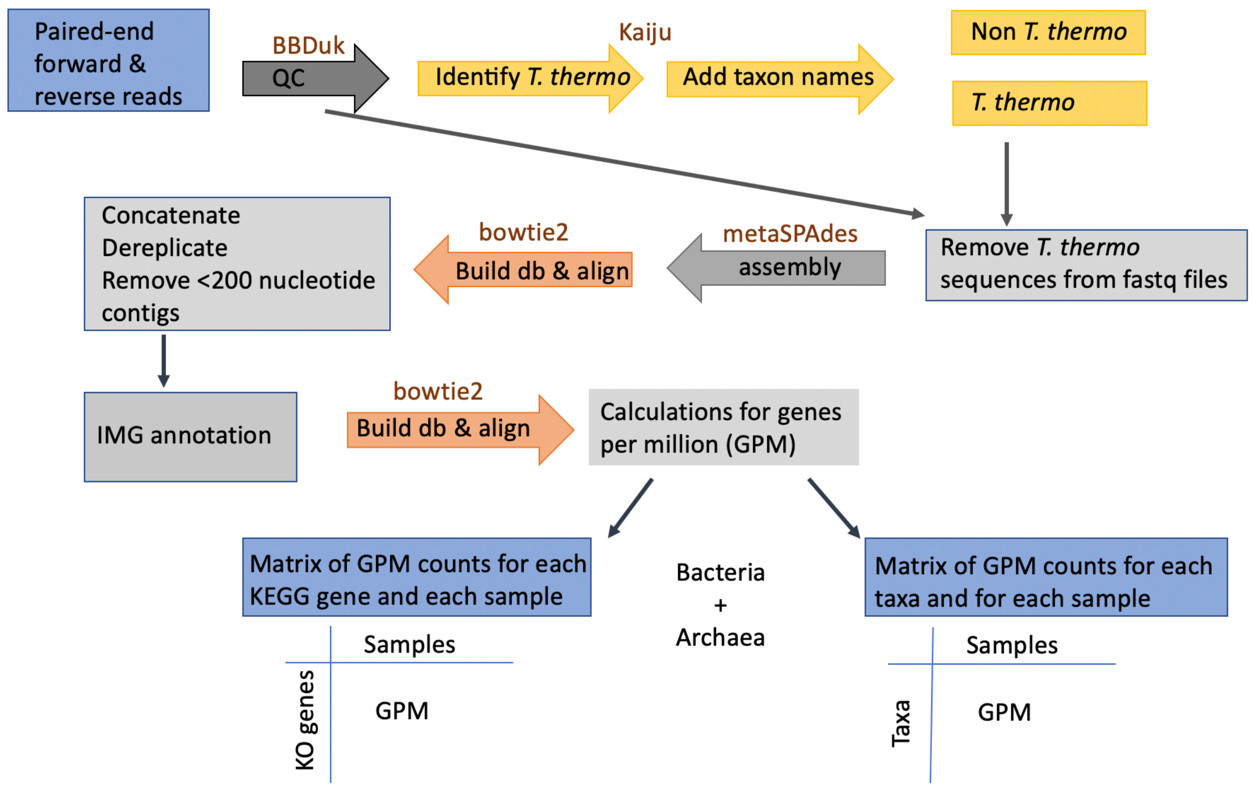

Supplementary Figure S1: Bioinformatics pipeline workflow. Bioinformatics software used is indicated in red text above arrows.

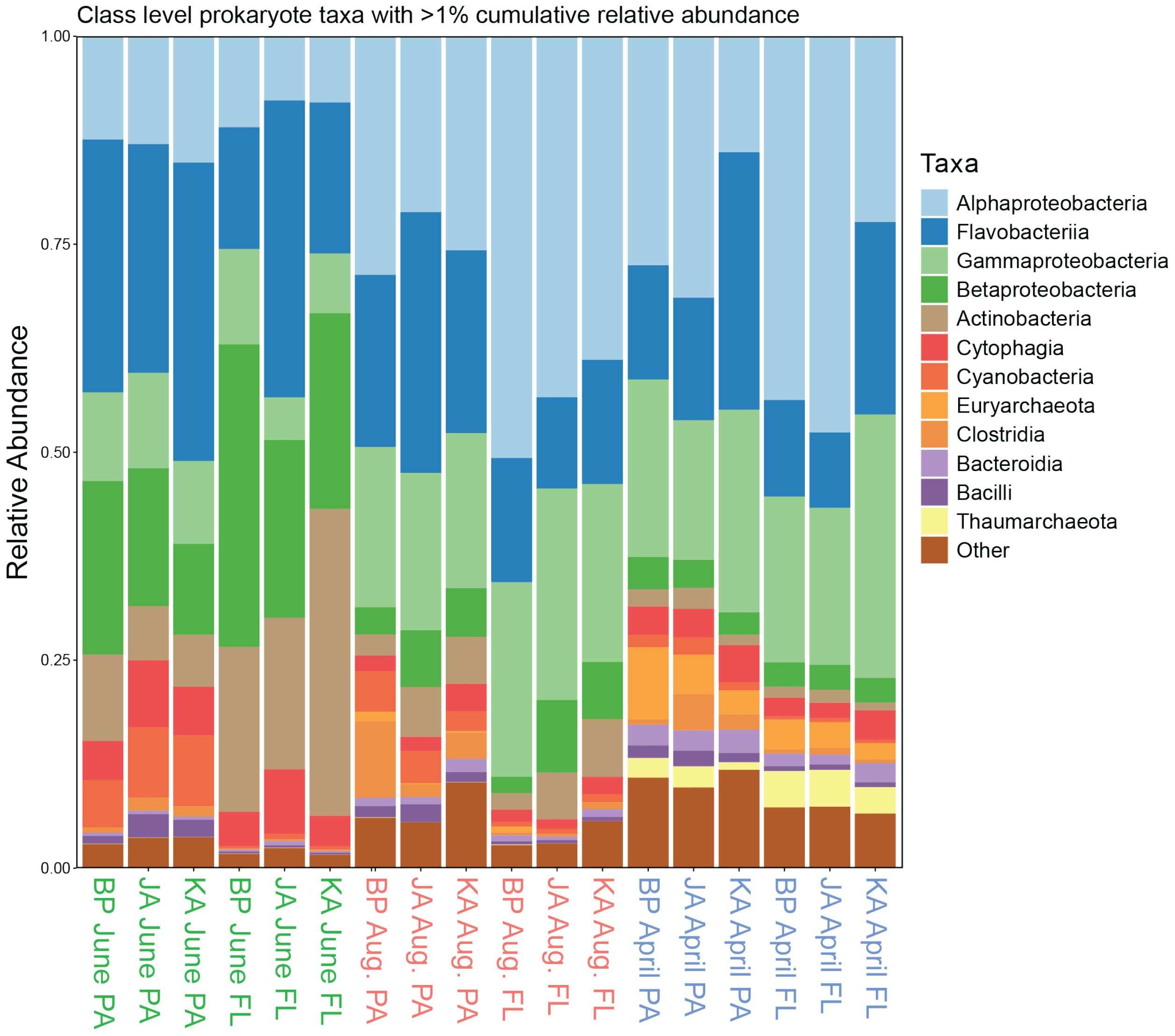

Supplementary Figure S2: Relative abundance of Bacteria and Archaea at the class level based on taxonomic assignments of KEGG-annotated genes. Taxa with <1% cumulative relative abundance are grouped as "Other".

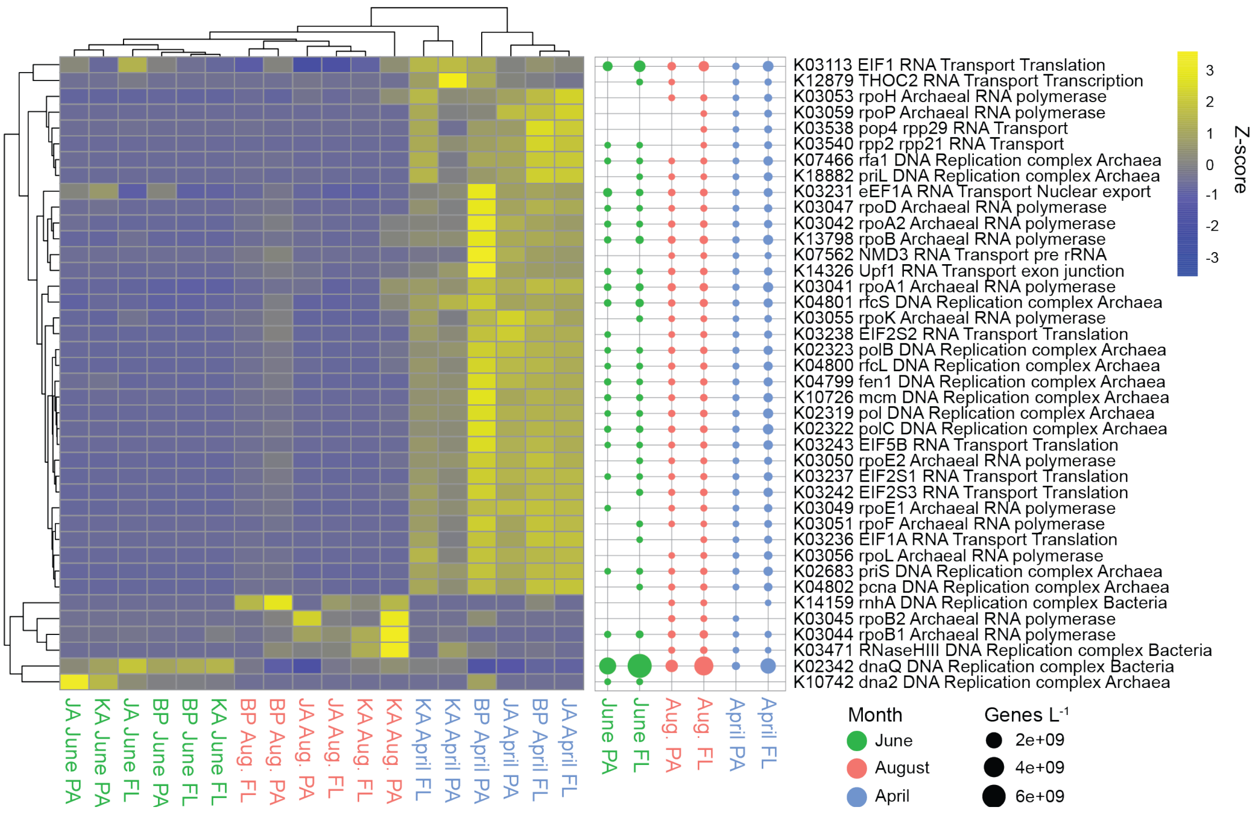

Supplementary Figure S3: DNA and RNA processing indicator genes. Heatmap of z-scaled GPM gene abundances for each sample with yellow being higher abundance and blue lower. Bubble plot of genes/L abundance for each month and size-fraction pairing.

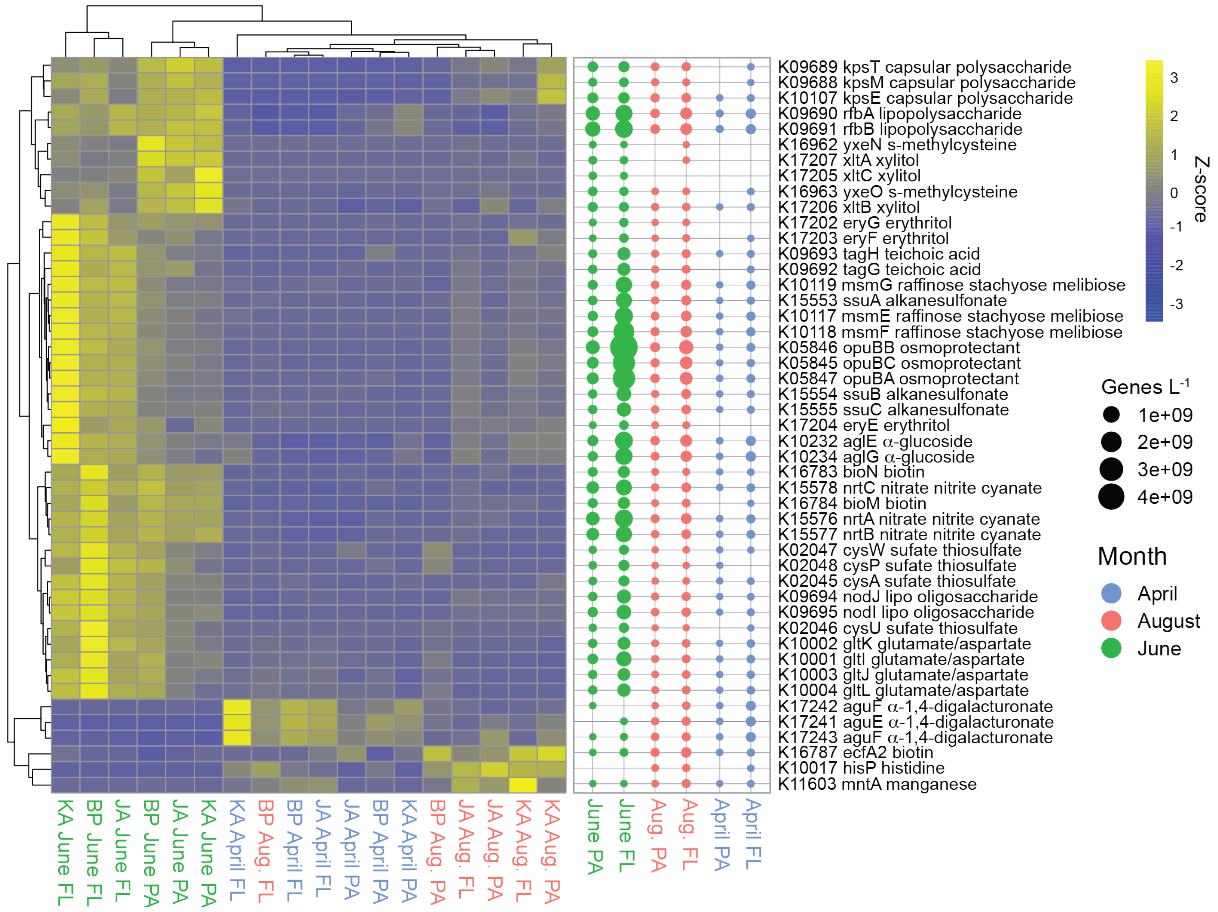

Supplementary Figure S4: ABC Transporter indicator genes (KEGG pathway ko02010). Heatmap of z-scaled GPM gene abundances for each sample with yellow being higher abundance and blue lower. Bubble plot of genes/L abundance for each month and size-fraction pairing.

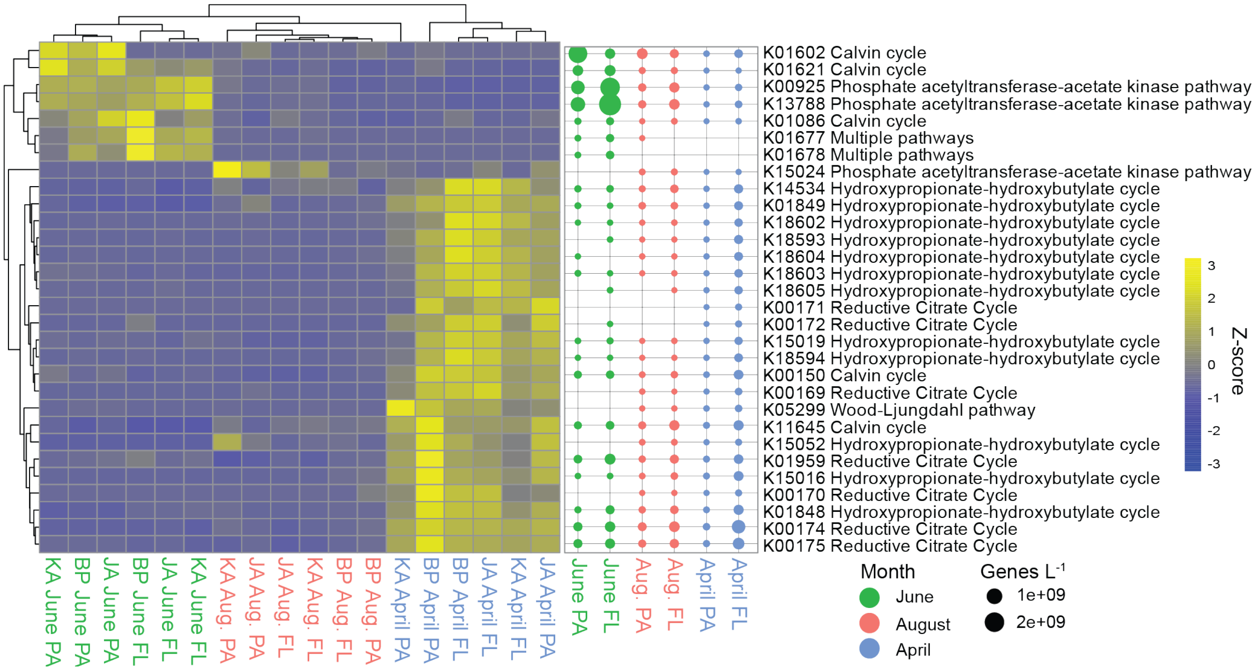

Supplementary Figure S5: Carbon fixation indicator genes from the KEGG pathways Carbon fixation in prokaryotes (map00720) and Carbon fixation in photosynthetic organisms (map00710). Heatmap of z-scaled GPM gene abundances for each sample with yellow being higher abundance and blue lower. Bubble plot of genes/L abundance for each month and size-fraction pairing.

Supplementary Tables

Supplementary Table S1. Metadata for each DNA sample including information about internal control addition of *Thermus thermophilus* HB-8 genomic DNA prior to extraction, recovery of T. thermophilus sequences, and calculation of multipliers to calculate genes per liter from metagenomic data following Satinsky et al. (2013). The following tabs provide the number of mapped reads (Rg), the length-corrected number of mapped reads scaled to the original number of reads (Tg-scaled), the genes-per-million relative abundance (GPM), and the genes per liter absolute abundance (genes-per-liter) for each KEGG Orthology gene number (KO) and taxomomy string (phylo). These abundance tables only include reads that were classified as prokaryotes (bacteria and archaea).

Supplementary Table S2. Location and environmental data associated with metagenome samples.

Supplementary Table S3. The number of indicator genes in KEGG pathways for each sampling period identified with the function multipatt (multi-level pattern analysis) in the indicspecies package available through R.

Supplementary Table S4. Information on individual coding sequences (CDS) that were annotated to mobility and chemotaxis genes belonging to the KEGG reference pathway "Two Component System" (map02020) including phylogenetic and functional (KEGG) annotations, and transcripts per million (TPM) of raw DNA sequences that mapped to each CDS.

Supplementary Table S5. Information on individual coding sequences (CDS) that were annotated to anoxygenic photosynthesis genes belonging to the KEGG reference pathway "Two Component System" (map02020) including phylogenetic and functional (KEGG) annotations, and transcripts per million (TPM) of raw DNA sequences that mapped to each CDS.

Supplementary Table S6. Information on individual coding sequences (CDS) that were annotated to nucleotide processing genes belonging to the KEGG reference pathways "DNA replication" (map03030), "RNA polymerase" (map03020), and "RNA transport" (map03010) including phylogenetic and functional (KEGG) annotations, and transcripts per million (TPM) of raw DNA sequences that mapped to each CDS.

Supplementary Table S7. Information on individual coding sequences (CDS) that were annotated to nitrification genes belonging to the KEGG reference pathway "Nitrogen metabolism" (map00910) including phylogenetic and functional (KEGG) annotations, and transcripts per million (TPM) of raw DNA sequences that mapped to each CDS.

Supplementary Table S8. Information on individual coding sequences (CDS) that were annotated to assimilatory and dissimilatory nitrate reduction genes belonging to the KEGG reference pathway "Nitrogen metabolism" (map00910) including phylogenetic and functional (KEGG) annotations, and transcripts per million (TPM) of raw DNA sequences that mapped to each CDS.

Supplementary Table S9. Information on individual coding sequences (CDS) that were annotated to denitrification genes belonging to the KEGG reference pathway "Nitrogen metabolism" (map00910) including phylogenetic and functional (KEGG) annotations, and transcripts per million (TPM) of raw DNA sequences that mapped to each CDS.

Supplementary Table S10. Information on individual coding sequences (CDS) that were annotated to photosynthesis genes belonging to the KEGG reference pathway "Photosynthesis" (map00195) including phylogenetic and functional (KEGG) annotations, and transcripts per million (TPM) of raw DNA sequences that mapped to each CDS.

Supplementary Table S11. Information on individual coding sequences (CDS) that were annotated to the dye decolorizing peroxidase gene (K15733) including phylogenetic and functional (KEGG) annotations, and transcripts per million (TPM) of raw DNA sequences that mapped to each CDS.

Supplementary Table S12. Information on individual coding sequences (CDS) that were annotated to genes belonging to the KEGG reference pathway "Methane metabolism" (map00680) including phylogenetic and functional (KEGG) annotations, and transcripts per million (TPM) of raw DNA sequences that mapped to each CDS.

PERL scripts

contig_regions_new.pl

This PERL script uses two files that are produced by the IMG annotation pipeline (Huntemann et al. 2016) to produce a fasta file of CDS sequences extracted from a fasta file of contig sequences.
1. A fasta file of contig sequences
2. A reformatted ".gff" file, which is a file produced by the IMG annotation pipeline containing information about each CDS sequence identified in a set of contigs. The .gff file from IMG must be modified before running this perl script so that it contains only the rows representing CDS (e.g., no tRNA or rRNA rows), and so that the last column contains only the CDS names.

Example command format:
contig_regions_new.pl -n 3300024273.a.CDS.gff -c 3300024273.a.fna -o 3300024273.CDS.fasta

```
#!/usr/bin/perl
###########################################################
#  CASHX
#
#  Copyright 2020
#
#  Sarah G. Nalven
#  Christopher M. Sullivan
#  Byron C. Crump
#
#  College of Earth, Ocean, and Atmospheric Sciences
#  Center for Genome Research and Biocomputing
#  Oregon State University
#  Corvallis, OR 97331
```

```
#
#  Byron.Crump@oregonstate.edu
#
# This program is not free software; you cannot redistribute it and/or
# modify it at all.
#
# This program is distributed in the hope that it will be useful,
# but WITHOUT ANY WARRANTY; without even the implied warranty of
# MERCHANTABILITY or FITNESS FOR A PARTICULAR PURPOSE.
#
########################################################
#
# Copyright (c) 2020 Oregon State University
# All Rights Reserved.
#
# Permission to use, copy, modify, and distribute this software and its
# documentation for educational, research and non-profit purposes, without
# fee, and without a written agreement is hereby granted, provided that
# the above copyright notice, this paragraph and the following three
# paragraphs appear in all copies.
#
# Permission to incorporate this software into commercial products may
# be obtained by contacting Oregon State University Office of Technology Transfer.
#
# This software program and documentation are copyrighted by Oregon State
# University. The software program and documentation are supplied "as is",
# without any accompanying services from Oregon State University. OSU does
# not warrant that the operation of the program will be uninterrupted or
# error-free. The end-user understands that the program was developed for
# research purposes and is advised not to rely exclusively on the program
# for any reason.
#
# IN NO EVENT SHALL OREGON STATE UNIVERSITY BE LIABLE TO ANY PARTY FOR
# DIRECT, INDIRECT, SPECIAL, INCIDENTAL, OR CONSEQUENTIAL DAMAGES,
# INCLUDING LOST PROFITS, ARISING OUT OF THE USE OF THIS SOFTWARE AND ITS
# DOCUMENTATION, EVEN IF OREGON STATE UNIVERSITYHAS BEEN ADVISED OF THE
# POSSIBILITY OF SUCH DAMAGE. OREGON STATE UNIVERSITY SPECIFICALLY
# DISCLAIMS ANY WARRANTIES, INCLUDING, BUT NOT LIMITED TO, THE IMPLIED
# WARRANTIES OF MERCHANTABILITY AND FITNESS FOR A PARTICULAR PURPOSE AND
# ANY STATUTORY WARRANTY OF NON-INFRINGEMENT. THE SOFTWARE PROVIDED
# HEREUNDER IS ON AN "AS IS" BASIS, AND OREGON STATE UNIVERSITY HAS NO
# OBLIGATIONS TO PROVIDE MAINTENANCE, SUPPORT, UPDATES, ENHANCEMENTS, OR
# MODIFICATIONS.
#
#########################################################

########################################################
# contig_regions_new.pl                                #
########################################################

use strict;
use warnings;
use Carp;
use Getopt::Std;
use Cwd;
use File::Copy;
use vars qw/ $opt_n $opt_c $opt_o $opt_h/;

########################################################
# Start Variable declarations                          #
########################################################
```

```
my ($id, $seq, $namefile, $configfile, $outfile);
my @seqarray;
my %seqhash;
my $totalseqs = 0;
my $totalfound = 0;

##########################################################
# End Variable declarations                             #
##########################################################

##########################################################
# Start Main body of Program                            #
##########################################################

 &getopts('vn:c:o:');

 &var_check();

 print "\n  Beginning Run:\n\n";
 print "\tStarting to process configs file...\n";

 open (OUT, "> $outfile") || die("Can not open outfile!");
 open (DAT, $configfile) || die("Can not open file!");
 while (<DAT>) {
       my $line  = $_;
             if($line =~ m/>/) {
                          if($totalseqs) {
                          $seq =~ s/\r//g;
                          $seq =~ s/\n//g;
                                       my %hash;
                          $hash{'seq'} = $seq;
                                       $seqhash{$id} = \%hash;
                          }
                          $seq = '';
                          $id = $_;
             $id  =~ s/\r//g;
             $id  =~ s/\n//g;
             $id  =~ s/>//g;
                          $totalseqs++;
             }
             else {
                          $seq .= $_;

             }

 }

 $seq =~ s/\r//g;
 $seq =~ s/\n//g;
 my %hash;
 $hash{'seq'} = $seq;
 $seqhash{$id} = \%hash;
 $totalseqs++;

 print "\tStarting to process names file...\n";


 open (DAT2, $namefile) || die("Can not open file!");
 while (<DAT2>) {
                          $_  =~ s/\r//g;
                          $_  =~ s/\n//g;
                          my ($name_id, $FGMP, $CDS, $start, $stop, $chrom, $strand, $score, $anno) = split(/\t/, $_);
```

```
                        if(defined($seqhash{$name_id})){
                        my $newhash = $seqhash{$name_id};
                                    my $seq = $newhash->{'seq'};
                                    my $subseq = substr($seq, ($start-1), (($stop-$start)+1));
                                    print OUT ">$anno\n$subseq\n";
                                    $totalfound++;
                        }
}
```

rl_wrapper.pl

This perl script extracts information from a .sam file that is produced by mapping a dataset of paired-end DNA sequences to a database of assembled CDS sequences using Bowtie2. This script was used to extract the basepair length of each CDS (cds.length), the number of reads mapped to each CDS (n.reads), and the average length of reads that mapped to each CDS (rl). It also multiplies n.reads by rl (ra.g). Note that this script counts forward and reverse reads separately.

Example command format:
perl /nfs1/Crump_Lab/bin/rl_wrapper.pl -i $(DIRPATH)/$(TAXONID).CDS.sam -o $(DIRPATH)/$(TAXONID).CDS.rl.tab

Dependencies:
This script uses the shell script pileup.sh, which is part of BBTools (Bushnell 2014)

```
#!/usr/src/perl -w
#############################################################
#  CASHX
#
#  Copyright 2020
#
#  Thomas J. Sharpton
#  Sarah G. Nalven
#  Byron C. Crump
#
#  Department of Microbiology
#  College of Earth, Ocean, and Atmospheric Sciences
#  Center for Genome Research and Biocomputing
#  Oregon State University
#  Corvallis, OR 97331
#
#  Byron.Crump@oregonstate.edu
#
# This program is not free software; you cannot redistribute it and/or
# modify it at all.
#
# This program is distributed in the hope that it will be useful,
# but WITHOUT ANY WARRANTY; without even the implied warranty of
# MERCHANTABILITY or FITNESS FOR A PARTICULAR PURPOSE.
#
#############################################################
#
# Copyright (c) 2020 Oregon State University
# All Rights Reserved.
#
```

```
# Permission to use, copy, modify, and distribute this software and its
# documentation for educational, research and non-profit purposes, without
# fee, and without a written agreement is hereby granted, provided that
# the above copyright notice, this paragraph and the following three
# paragraphs appear in all copies.
#
# Permission to incorporate this software into commercial products may
# be obtained by contacting Oregon State University Office of Technology Transfer.
#
# This software program and documentation are copyrighted by Oregon State
# University. The software program and documentation are supplied "as is",
# without any accompanying services from Oregon State University. OSU does
# not warrant that the operation of the program will be uninterrupted or
# error-free. The end-user understands that the program was developed for
# research purposes and is advised not to rely exclusively on the program
# for any reason.
#
# IN NO EVENT SHALL OREGON STATE UNIVERSITY BE LIABLE TO ANY PARTY FOR
# DIRECT, INDIRECT, SPECIAL, INCIDENTAL, OR CONSEQUENTIAL DAMAGES,
# INCLUDING LOST PROFITS, ARISING OUT OF THE USE OF THIS SOFTWARE AND ITS
# DOCUMENTATION, EVEN IF OREGON STATE UNIVERSITYHAS BEEN ADVISED OF THE
# POSSIBILITY OF SUCH DAMAGE. OREGON STATE UNIVERSITY SPECIFICALLY
# DISCLAIMS ANY WARRANTIES, INCLUDING, BUT NOT LIMITED TO, THE IMPLIED
# WARRANTIES OF MERCHANTABILITY AND FITNESS FOR A PARTICULAR PURPOSE AND
# ANY STATUTORY WARRANTY OF NON-INFRINGEMENT. THE SOFTWARE PROVIDED
# HEREUNDER IS ON AN "AS IS" BASIS, AND OREGON STATE UNIVERSITY HAS NO
# OBLIGATIONS TO PROVIDE MAINTENANCE, SUPPORT, UPDATES, ENHANCEMENTS, OR
# MODIFICATIONS.
#
############################################################

use strict;
use Getopt::Long;

my( $in_file, $pileup_outfile,
   $genelen_outfile, $output );
my $rl_wrapper_path = "/nfs1/Crump_Lab/bin/";

GetOptions(
   "i=s"   => \$in_file,
   "po=s"  => \$pileup_outfile,
   "glo=s" => \$genelen_outfile,
   "o=s"   => \$output, #the final output file
   "p:s"   => \$rl_wrapper_path #where to find this and related scripts
   );

#check input variables
if( !defined( $in_file ) ){
   die "You must specify a .sam file to process " .
         "via option -i";
}
if( !defined( $output ) ){
   die "You must specify an output table location " .
         "via option -o";
}
if( !defined( $pileup_outfile ) ){
   $pileup_outfile = "./pileup-tmp";
}
if( !defined( $genelen_outfile ) ){
   $genelen_outfile = "./genelen-tmp";
}
```

```
#run the workflow
`pileup.sh in=${in_file} out=${pileup_outfile}`;
_parse_gene_lengths_from_pileup( $pileup_outfile, $genelen_outfile );
`${rl_wrapper_path}calculate_rl_v2.pl -i ${in_file} -o ${output} -t ${genelen_outfile}`;

#####
# SUBROUTINES
#####

sub _parse_gene_lengths_from_pileup{
   my $pileup_out  = shift;
   my $genelen_out = shift;
   open( IN, $pileup_out ) ||
            die "Can't open $pileup_out for read: $!\n";
   open( OUT, ">$genelen_out" ) ||
            die "Can't open $genelen_out for write: $!\n";
   while(<IN>){
            chomp $_;
            my @data = split( "\t", $_);
            my $gene = $data[0];
            my $len  = $data[2];
            print OUT "${gene}\t${len}\n";
   }
   close IN;
   close OUT;
   return;
}
```

References

Bushnell, B., 2014. BBMap: a fast, accurate, splice-aware aligner (No. LBNL-7065E). Lawrence Berkeley National Lab (LBNL), Berkeley, CA (United States).

Gregg, F., and Eder, D. (2019). Dedupe. Available at: https://github.com/dedupeio/dedupe.

Huntemann, M., Ivanova, N. N., Mavromatis, K., Tripp, H. J., Paez-Espino, D., Tennessen, K., et al. (2016). The standard operating procedure of the DOE-JGI Metagenome Annotation Pipeline (MAP v.4). *Stand Genomic Sci* 11, 17. doi:10.1186/s40793-016-0138-x.

Langmead, B., and Salzberg, S. L. (2012). Fast gapped-read alignment with Bowtie 2. *Nature Methods* 9, 357–359. doi:10.1038/nmeth.1923.

Menzel, P., Ng, K. L., and Krogh, A. (2016). Fast and sensitive taxonomic classification for metagenomics with Kaiju. *Nature Communications* 7. doi:10.1038/ncomms11257.

Nurk, S., Meleshko, D., Korobeynikov, A., and Pevzner, P. A. (2017). metaSPAdes: a new versatile metagenomic assembler. *Genome Res.* 27, 824–834. doi:10.1101/gr.213959.116.

O'Leary, N. A., Wright, M. W., Brister, J. R., Ciufo, S., Haddad, D., McVeigh, R., et al. (2016). Reference sequence (RefSeq) database at NCBI: current status, taxonomic expansion, and functional annotation. *Nucleic Acids Res.* 44, D733-745. doi:10.1093/nar/gkv1189.

Satinsky, B. M., Gifford, S. M., Crump, B. C., and Moran, M. A. (2013). "Use of Internal Standards for Quantitative Metatranscriptome and Metagenome Analysis," in *Methods in Enzymology* (Elsevier), 237–250. doi:10.1016/B978-0-12-407863-5.00012-5.

Wagner, G. P., Kin, K., and Lynch, V. J. (2012). Measurement of mRNA abundance using RNA-seq data: RPKM measure is inconsistent among samples. *Theory in Biosciences* 131, 281–285. doi:10.1007/s12064-012-0162-3.
